# A Rare Case Report of Neuroimaging, Electrophysiological, and Dynamic Laryngoscopy Studies in a Stroke Patient of Foix‐Chavany‐Marie Syndrome

**DOI:** 10.1002/ccr3.72438

**Published:** 2026-04-15

**Authors:** Lu Xia, Liyan Cai, Xin Chen, Wei Huang, Cen Chen, Shinian Yang, Qing Chen, Yuanyuan Zhu

**Affiliations:** ^1^ Department of Rehabilitation Chengdu First People's Hospital Chengdu Sichuan China; ^2^ Department of Rehabilitation Chengdu Integrated TCM&Western Medicine Hospital Chengdu Sichuan China; ^3^ Department of Neurology Chengdu First People's Hospital Chengdu Sichuan China; ^4^ Department of Neurology Chengdu Integrated TCM&Western Medicine Hospital Chengdu Sichuan China; ^5^ Department of Otolaryngology Chengdu First People's Hospital Chengdu Sichuan China; ^6^ Department of Otolaryngology Chengdu Integrated TCM&Western Medicine Hospital Chengdu Sichuan China

**Keywords:** apraxia of eyelid closure, blink reflex, dysphagia, Foix‐Chavany‐Marie syndrome, ischemic stroke, laryngoscopy

## Abstract

Foix‐Chavany‐Marie Syndrome (FCMS), also known as opercular syndrome, is a rare neurological disorder caused by an ischemic stroke that results in autonomic‐voluntary dissociation. In this case report, we describe a patient who presented with an acute right frontal and parietal cortical lesion, in addition to a remote infarction in the left lateral paraventricular region. The patient exhibited a masked face, facial paralysis, dysarthria, dysphagia, and apraxia of eyelid closure while automatic pathways, such as crying and laughing, remained intact. MRI revealed an acute ischemic lesion in the operculum and a chronic contralateral lesion. Blink reflex testing indicated intact brainstem pathways but an impaired corticobulbar tract. Dynamic laryngoscopy showed severe impairment in the oral and pharyngeal stages of swallowing. Early diagnosis and a multimodal therapeutic approach, including acupuncture and swallowing training, significantly improved patient outcomes, including the resolution of eyelid motor control deficits. The patient had a relatively good prognosis.

**Key Clinical Message**

This FCMS case, resulting from acute and prior contralateral corticonuclear tract injuries, presented with severe dysphagia but showed good recovery. Blink reflex results indicated intact brainstem pathways, while the corticobulbar tract was impaired. Dynamic laryngoscopy showed severe impairment in the oral and pharyngeal stages of swallowing.

## 1 Introduction

Opercular syndrome, or Foix‐Chavany‐Marie syndrome (FCMS), is a rare form of cortical pseudobulbar paralysis characterized by paralysis of voluntary movements in the face, tongue, throat, and masticatory muscles. Notably, reflex and automatic movements remain intact in these regions, a phenomenon termed “automatic‐voluntary dissociation”. It was first described by German doctor Magnus in 1837 [1] and later officially reported and named in 1926 by the French doctor Foix [2]. Patients typically exhibited a distinctive facial appearance, characterized by a predominantly expressionless countenance with a partially open mouth accompanied by persistent salivation, along with facial paralysis, dysarthria, and dysphagia. The voluntary movements of the facial, tongue, and pharyngeal muscles are mediated by the primary motor cortex and descending pyramidal tracts, while involuntary movements and emotional responses are regulated by the thalamus, hypothalamus, and extrapyramidal system. Thus, structural or functional disruption of the neural pathways that connect the bilateral cortical motor areas to the brainstem nuclei is hypothesized to underlie the autonomic‐voluntary motor dissociation observed in FCMS [3, 4].

In this case study, we reported a patient presenting with a distinctive facial phenotype, characterized by a predominantly expressionless countenance, alongside facial paralysis, dysarthria, dysphagia, and apraxia of eyelid closure, which collectively met the diagnostic criteria for FCMS. Neuroimaging revealed the responsible lesion focus as an acute right frontal and parietal cortical lesion and remote left lateral paraventricular region infarction. Additionally, we performed the first‐ever documented blink reflex assessment and dynamic laryngoscopy in this FCMS patient. Early diagnosis combined with a multimodal therapeutic regimen, including acupuncture, swallowing training, and targeted rehabilitation, might substantially enhance functional outcomes in FCMS.

## 2 Clinic History/Examination

A 60‐year‐old man presented to our hospital with symptoms of left‐sided limb weakness and slurred speech lasting over 7 h. Approximately 7 h prior to admission, he developed slurred speech, pseudobulbar affect, left‐sided limb weakness, and difficulty gripping objects and maintaining balance, with no apparent trigger. These symptoms were accompanied by sensory loss in the left limb and dizziness. He was promptly taken to the emergency department, where he underwent a head CT scan. The scan revealed subtle hypodensity in the left lateral paraventricular region and right centrum semiovale, suggestive of acute cerebral infarction. Since symptom onset, he has remained in a stable mood, with normal appetite and regular bowel movements, although he reported poor sleep quality. No significant weight loss was documented. His medical history includes an ischemic stroke in 2016, which resulted in mild right‐sided hemiparesis, and muscle tone in the right lower limb was slightly increased. Atherosclerosis is likely the primary etiology of the patient's previous stroke, as evidenced by MRA findings demonstrating stenosis in the left anterior and middle cerebral arteries. Notably, the patient had not received secondary stroke prevention measures. He has a history of hypertension, diagnosed six months ago. His condition is managed with a daily regimen of 5 mg amlodipine, which has maintained good blood pressure control. Additionally, he underwent a cholecystectomy in 2017 for gallbladder stones.

Upon admission, the patient's vital signs were within the following ranges: Temperature (T) 36.4°C; pulse (P) 96 beats/min; respiratory rate 19 breaths/min; blood pressure (BP) 150/100 mmHg. The neurological assessment demonstrated an alert and oriented mental status, appropriate responses, and fluent cooperation. However, mild dysarthria was observed during speech evaluation. The examination also revealed intact orientation to time, place, and person. The cognitive assessment revealed preserved calculation ability and short‐term memory recall. Cranial nerve testing showed left‐sided upper motor neuron facial weakness. Pupils were isochoric (3 mm bilaterally), round, and sensitive to light reflection. Ocular motility remained intact, with a full range of motion and absence of nystagmus. Cranial nerve examination demonstrated midline tongue position at rest, with examination revealing dysarthria and dysphagia. Higher cortical functions showed no evidence of constructional or ideomotor apraxia, with preserved left–right orientation. Muscle tone in the right lower limb slightly increased, while the rest had normal muscle tone. Muscle strength testing showed left‐sided hemiparesis (Grade 4/5 on the Manual Muscle Testing), contrasting with normal right‐sided strength (Grade 5/5). The sensory evaluation identified diminished light touch and pinprick sensation on the left side; no pathological signs were present bilaterally. The mRS score was 4, indicating severe disability requiring assistance for self‐care. Cognitive assessment using the Mini‐Mental State Examination (MMSE) yielded a score of 27/30, falling within the normal range. Standardized swallowing assessment (SSA) results showed a score of 23/46, corresponding to grade 1 (light) aspiration risk.

**FIGURE 1:**
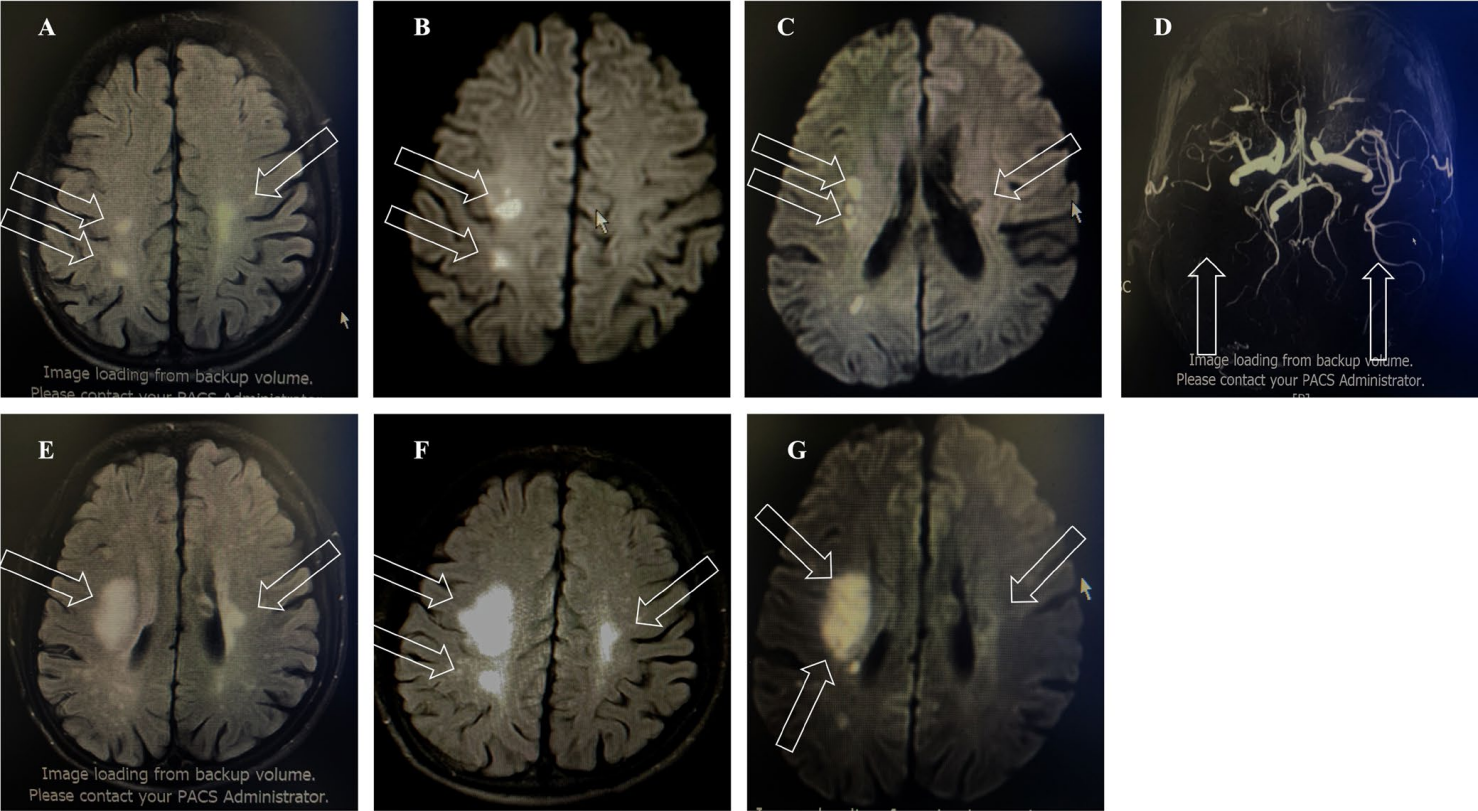
Neuroimaging features of the patient after two strokes. Axial T2‐FLAIR and DWI imaging of the patient with stroke. The patient demonstrated an acute stroke in the right parietal lobe and a remote stroke on the left centrum semiovale on the T2‐FLAIR window (A) and DWI imaging window (B, C) at the time of the first stroke. The MRA shows occlusion of the lower trunk of the right middle cerebral artery M2 segment, with a sparse representation of the distal branches of the right middle cerebral artery (D). The acute stroke on the right frontal and parietal lobes and remote stroke on the left centrum semiovale on T2‐FLAIR window (E) and DWI imaging window (F, G) at the second time of stroke. The arrow showed the lesions in each picture.

Neuroimaging studies, including brain MRI and MRA, demonstrated a punctate acute infarct in the center of the frontal and parietal lobes, along with scattered ischemic areas and previous cerebral infarctions. MRA demonstrated occlusion of the lower trunk of the right middle cerebral artery M2 segment, with sparse representation of the distal branches of the right middle cerebral artery. MRI follow‐up at a 9‐day interval demonstrated significant progression of the lesion size compared to the initial scan (Figure 1). Combined with the medical history and auxiliary examination, the cerebral infarction was definitively attributed to the large artery atherosclerosis (LAA) subtype per the Trial of Org 10,172 in Acute Stroke Treatment (TOAST) classification.

**FIGURE 2:**
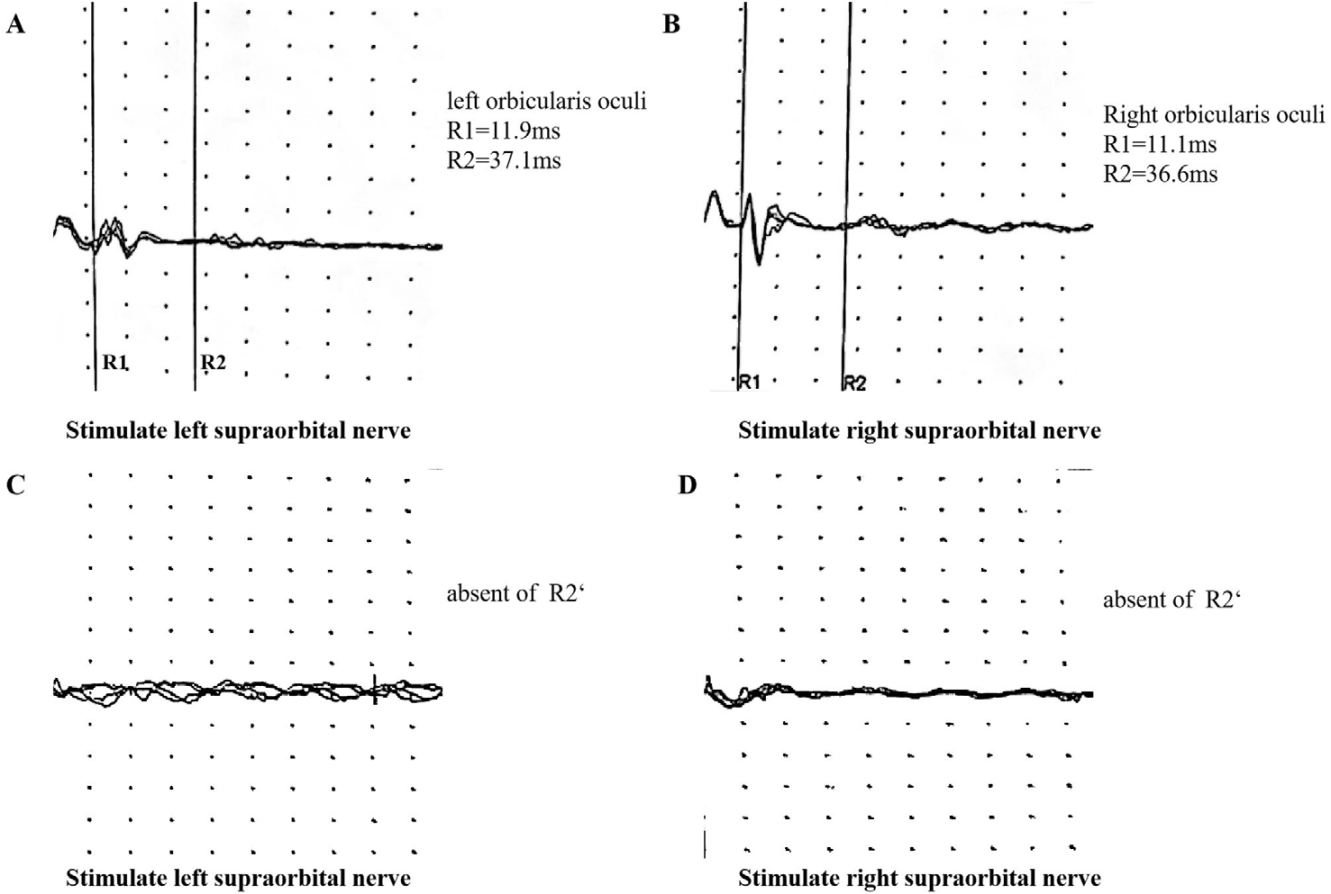
Blink reflex findings during the patient's eyelid movement disorder. Stimulation of the supraorbital nerve elicits normal R1 and R2 responses in the orbicularis oculi (OO) muscles (A, B), but demonstrates the absence of the R2′ response on bilateral sides (C, D).

For nearly one month after the stroke, the patient exhibited a specific symptom characterized by an inability to maintain eyelid closure for more than 1–3 s. Occasionally, he manually closed his eyelids, but they reopened immediately upon release. Notably, he retained the ability to blink spontaneously and maintain complete eyelid closure during sleep (see Supplementary Video S2). During the period of eyelid movement dysfunction, a blink reflex test was performed. The results demonstrated normal R1 and R2 responses in the orbicularis oculi (OO) muscles following supraorbital nerve stimulation, but the contralateral R2′ response was absent (Figure 2). The result indicated impaired cortical‐to‐brainstem signaling, but preserved brainstem nuclei‐to‐lower motor neuron function was observed.

**FIGURE 3:**
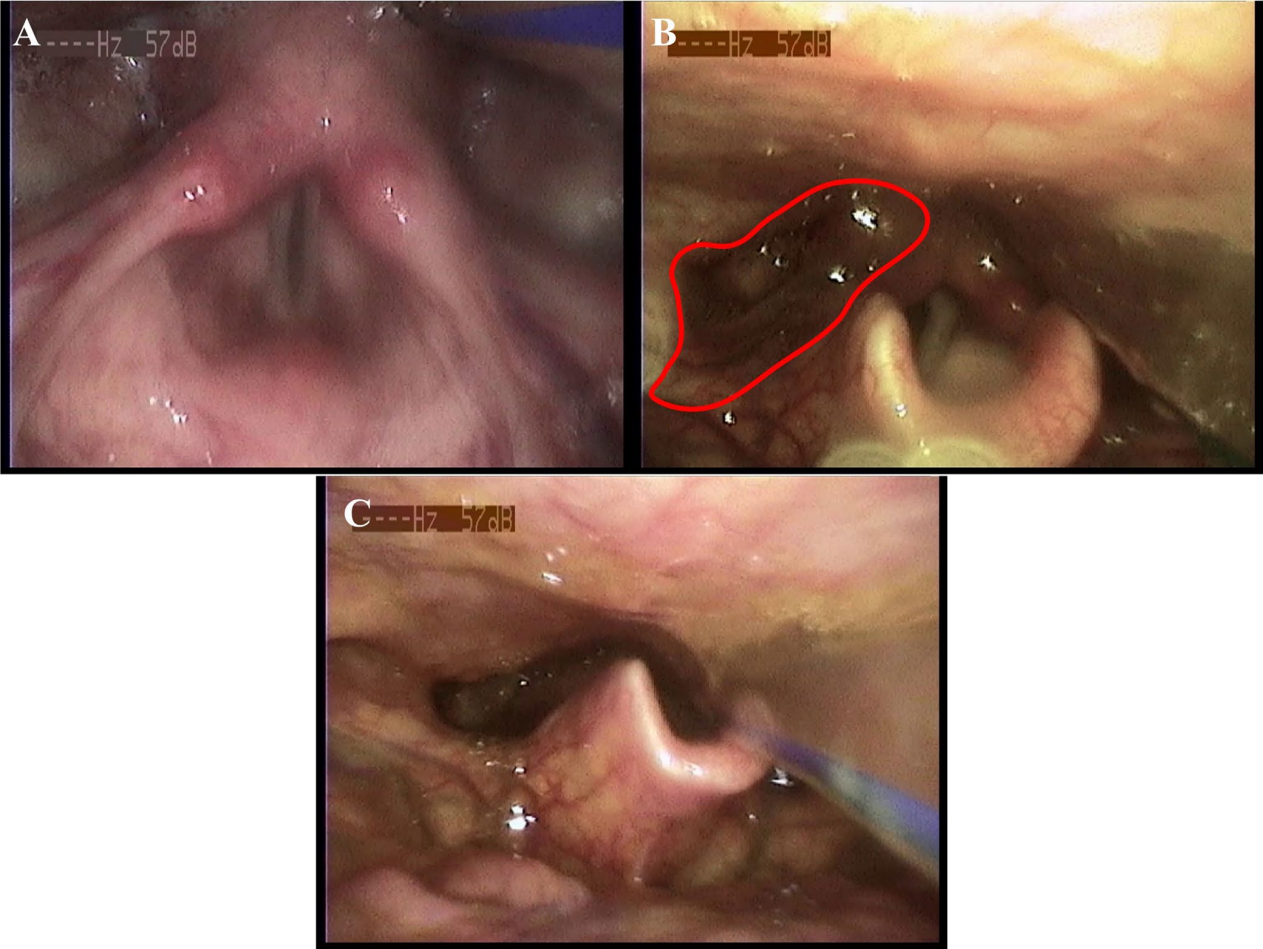
Dynamic laryngoscopy findings in the patient with oropharyngeal‐phase dysphagia. Localized hypopharyngeal hyperemia was observed. The bilateral vocal folds demonstrated smooth margins without significant hyperemia or edema. Left vocal fold slack with an arcuate closure pattern (A). Residue in the piriform sinuses (B). Clearance of the residue was achieved through repeated swallowing efforts (C).

Dynamic laryngoscopy revealed dysphagia during the oropharyngeal phase. The examination demonstrated localized hyperemia in the hypopharynx. No significant hyperemia or edema was observed in the bilateral vocal cords, which exhibited smooth edges. The left vocal fold appeared loose and displayed an arcuate closure pattern. Residual material was identified in the piriform sinuses. This residue was cleared following repeated swallowing efforts. Additionally, the patient exhibited delayed coughing following ingestion of 3 mL and 5 mL of low‐consistency food. Secretions surrounding the laryngeal vestibule were graded using the Murray Secretion Severity Scale (MSS), a four‐level rating scale used to determine the severity of accumulated oropharyngeal secretions during laryngoscopy. These secretions, when bilaterally present or forming deep pooling, were graded as level 1 [5]. Residual food material was observed in the epiglottic valleculae. The residue extending to the visible laryngeal surface of the epiglottis was scored as 3 points, while material filling the epiglottic margins and aryepiglottic folds was graded as 5 points according to the YPR‐SRS [6]. Aspiration risk was subsequently assessed using the 8‐point Penetration‐Aspiration Scale (PAS), scores are determined primarily by the depth to which material passes in the airway and by whether or not material entering the airway is expelled [7]. This sequence of events was classified as level 6 on the PAS, indicating laryngeal penetration below the vocal folds with aspiration, which could be completely cleared (Figure 3 and Video S1).

## 3 Diagnostic Criteria, Investigations, and Treatment

According to the three general classes of tests utilized by physicians when determining a diagnosis for FCMS: (1) The patient exhibited voluntary paralysis of the facial, lingual and pharyngeal muscles, while automatic pathways, such as those for crying and laughing, remained preserved; (2) Standardized swallowing assessment (SSA) results showed a score of 23/46, corresponding to grade 1 (light) aspiration risk. Psycholinguistic testing revealed that the patient has dysarthria and motor aphasia. (3) Neuropsychological testing with cognitive assessment using the Mini‐Mental State Examination (MMSE) yielded a score of 27/30, indicating normal cognition [8, 9]. This case was localized to the disruption of the corticonuclear tract connecting the precentral gyrus to the brainstem motor nuclei. The anterior opercular cortex, vascularized by the superior division of the middle cerebral artery (MCA), demonstrated acute ischemic damage on MRI, corroborated by MRA evidence of MCA‐M2 segment stenosis. In addition, neuroimaging revealed an acute ischemic stroke in the opercular region and a remote infarction in the left lateral paraventricular region. These findings confirmed the diagnosis of FCMS.

The patient's management strategy employed a multidisciplinary approach that systematically targeted acute symptom control, modulation of underlying pathophysiology, and prophylaxis against secondary complications. To address the cerebral infarction, physicians prescribed clopidogrel bisulfate tablets 75 mg once daily and aspirin enteric‐coated tablets 100 mg for dual antiplatelet therapy (DAPT) over 21 days, followed by monotherapy for secondary prevention. The medical team administers rosuvastatin calcium tablets 10 mg at night to lower lipids and stabilize plaque. Clinicians administer sodium butylphthalide chloride injection 100 mL intravenously every 12 h for 14 days to improve collateral circulation. They also infuse edaravone dextrol concentrated injection solution 15 mL every 12 h for 14 days to scavenge free radicals. Healthcare staff provided citicoline sodium tablets 0.2 g three times a day via tube feeding to support brain protection and nerve nutrition. They also administer rabeprazole sodium enteric‐coated tablets 20 mg at night to suppress acid and protect the stomach. For mucolytic management, clinicians injected ambroxol hydrochloride 15 mg intravenously every 12 h. The team gave zopiclone tablets 3 mg at night to improve insomnia. Physicians prescribe nifedipine sustained‐release tablets 20 mg and sacubitril valsartan sodium tablets 200 mg once daily as an antihypertensive regimen. They also administered levodopa tablets 125 mg three times a day to supplement dopamine.

**FIGURE 4:**
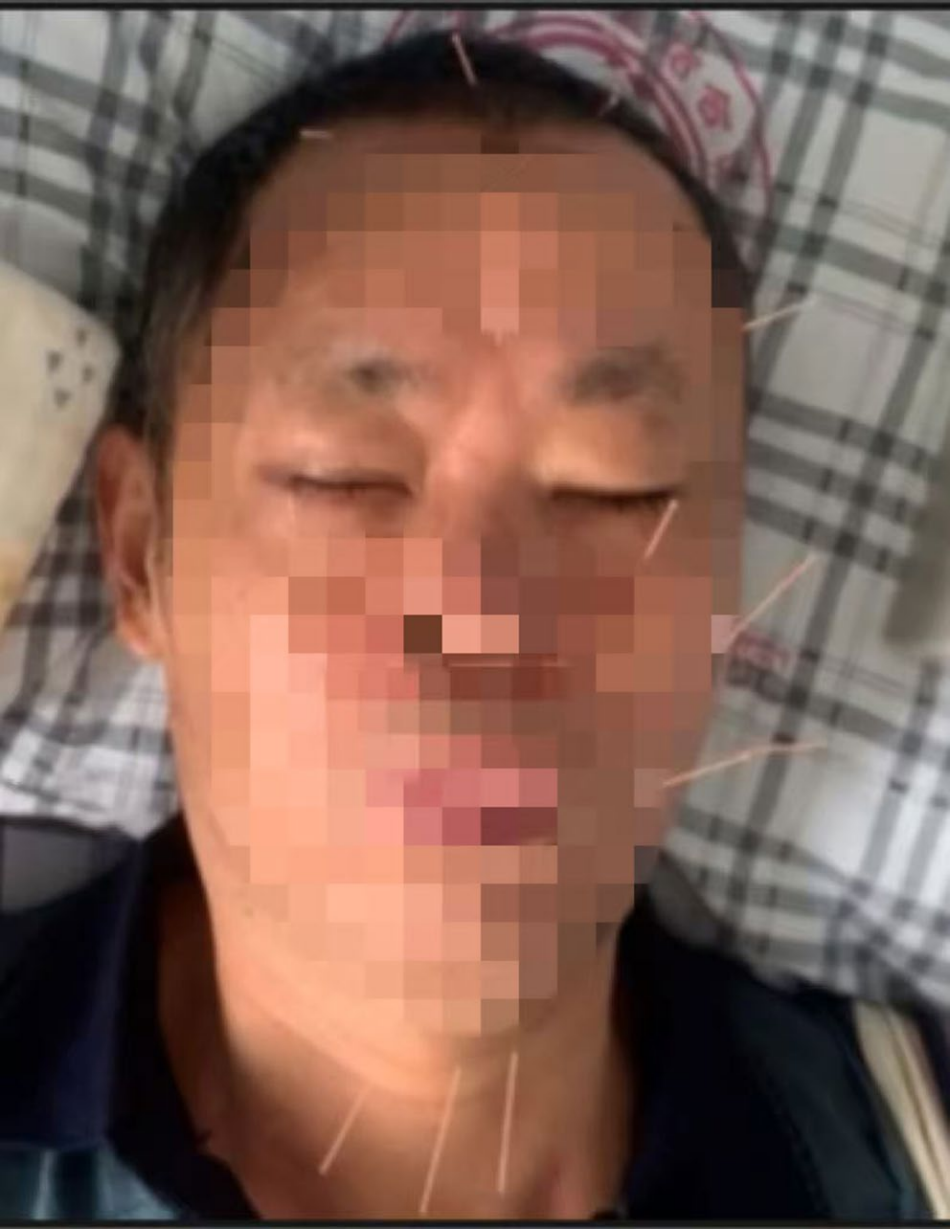
Location of acupoints with the stimulation of oral, facial, and swallowing functions.

**FIGURE 5:**
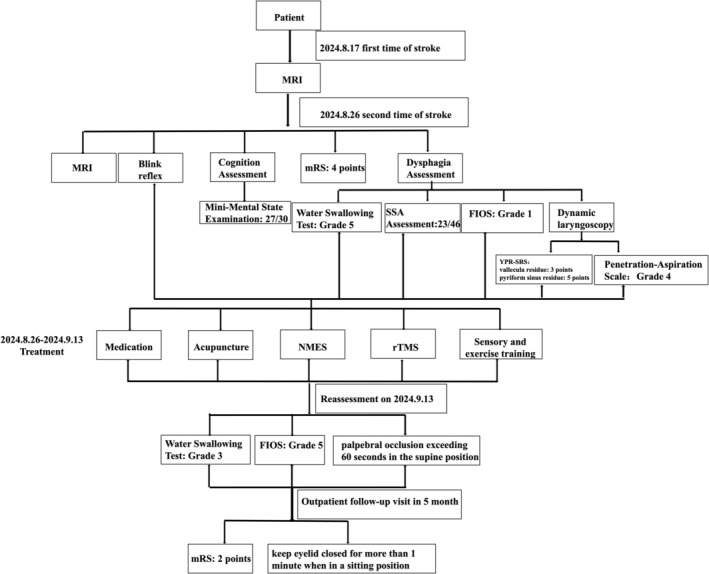
The flow chart of the study. The figure illustrates the patient's journey from the initial stroke onset to the 5‐month follow‐up. The initial MRI (August 17, 2024) confirmed the first stroke event. A second MRI (August 26, 2024) revealed a new location of the stroke. Subsequent multidisciplinary treatment, including medication, acupuncture, NMES, rTMS, and sensory and motor training, was administered. Reassessment on September 13, 2024, showed significant improvements in swallowing function (Water Swallowing Test from Grade 5 to 3, FIOS from Grade 1 to 5) and the ability to maintain eyelid closure in a supine position. At the 5‐month outpatient follow‐up, the patient demonstrated sustained clinical improvement with a modified Rankin Scale (mRS) score of 2 and the ability to keep eyelids closed for over one minute while sitting.

Due to clinically evident aspiration, enteral nutrition was initiated via a nasogastric tube. The patient underwent physical and occupational therapy for hemiplegia. The treatment team employed neuromuscular electrical stimulation therapy, behavioral interventions, acupuncture, and repetitive transcranial magnetic stimulation to treat dysphagia. Therapists applied behavioral interventions comprising management and treatment methods tailored to the patient's swallowing function. In addition, they recommended adjusting swallowing postures and using compensatory strategies (Table 1). Clinicians incorporated acupuncture and moxibustion into the treatment regimen to further improve facial and limb movement, dysphagia, and aphasia. For acupuncture therapy, they selected the following acupoints (see Figure 4 and Table 2) in accordance with WHO standards [8]. They used disposable acupuncture needles (0.18 mm × 25 mm) and inserted them to a depth of approximately 18–23 mm until achieving a deqi response. The practitioners performed each acupuncture session for 20 min, five times per week (Figure 5).

**TABLE 1:** The selection of acupoints. The acupoints of EX‐HN3, EX‐HN4, ST1, SIl8, ST4, DU24, BL4, RN23 were selected.

Acupoints	Location
Yintang (EX‐HN3)	On the forehead, midway between the eyebrows.
Yuyao (EX‐HN4, left side)	In the middle of the eyebrow. When looking straight ahead, the point is directly above the pupil.
Chengqi (ST1, both sides)	With the eyes looking straight ahead, the point is directly below the pupil, between the eyeball and the infraorbital ridge.
Quanliao (SIl8, left side)	On the face, directly below the lateral canthus, in the depression on the lower border of the bone.
Dicang (ST4, left side)	On the face, lateral to the corners of the mouth, directly below the pupil.
Shenting (DU24)	On the head, 0.5 cun superior to the anterior hairline, directly above the glabella.
Qucha (BL4, both sides)	On the head, 0.5 cun superior to the anterior hairline, 1.5 cun lateral to the midline.
Jin's Tongue Three Needles	The three points consist of Lianquan (RN23) and two bilateral points. Lianquan (RN23) is located on the anterior midline of the neck, above the Adam's apple, in the depression superior to the hyoid bone. The two accessory points are located 0.5 cun lateral to RN23.

**TABLE 2:** Treatment strategies for the patient.

Treatment Method	Instrument model	Treatment parameters
Physical therapy	Neuromuscular re‐education techniques, including Bobath, Brunnstrom, PNF techniques, balance and coordination training, gait training and ambulation	45 min/day, 5times/week
Occupational Therapy	Upper limb and hand function training, and activities of daily living training	45 min/day, 5times/week
Swallowing and speech training	Oral sensory stimulation, oropharyngeal muscle exercise and airway protection technique training	45 min/day, 5times/week
Neuromuscular electrical stimulation therapy	Electrode patches were placed below the hyoid bone and on the thyroid cartilage, with low frequency	20 min, 5 times per week
Acupuncture and electropuncture	Low frequency, dilatational wave	20 min, 5 times per week
Repetitive transcranial magnetic stimulation	Left frontal lobe: 600 pulse 5 Hz, Right frontal lobe: 1,200 pulse 1 Hz	5 times per week

## 4 Conclusions and Results

By treatment day 18, the patient manifested significant recovery in orbicularis oculi motor control. Command compliance showed marked enhancement, with sustained palpebral occlusion exceeding 60 s in the supine position, while persistent eyelid dysfunction was observed in the seated position. The patient demonstrated progressive swallowing recovery. Water Swallowing Tests (WST) documented improvement from grade 5 (severe impairment) to grade 3 (moderate impairment) following 18 days of targeted dysphagia therapy. This functional enhancement ultimately permitted the safe removal of the nasogastric feeding tube. He regained the ability to eat solid food orally without coughing and could drink, albeit with caution. The Functional Intraoral Scale (FIOS) improved from 1 to 5. At his 5‐month outpatient follow‐up, the patient could move around, eat independently, and keep his eyelid closed for more than 1 min while sitting. The mRS score reached 2 (see Supplementary Video S2).

## 5 Discussion

This case report describes a patient with bilateral strokes (acute right frontoparietal and chronic left paraventricular infarcts) presenting with classic FCMS features: facial weakness, dysarthria, dysphagia, and apraxia of eyelid closure with motor impersistence, while emotional expressions remained intact. MRI confirmed acute opercular involvement with contralateral chronic damage. Novel evaluations included blink reflex testing, which demonstrated impaired corticobulbar transmission with preserved brainstem pathways, and dynamic laryngoscopy, which revealed significant oral/pharyngeal swallowing deficits not detailed in previous FCMS literature. Early multidisciplinary intervention, including acupuncture and swallowing therapy, led to notable symptom improvement and a favorable outcome.

The opercular syndrome was typically associated with bilateral damage because the bilateral corticonuclear tracts simultaneously innervate the nuclei of the 5th, 7th, 9th, and 10th cranial nerves, while unilateral involvement rarely causes this syndrome. The responsible lesions were not limited to the opercular cortex but could involve any region between the bilateral primary motor cortex and the brainstem motor nuclei. Patients may present with bilateral opercular damage, unilateral opercular cortical involvement with contralateral subcortical abnormalities, or bilateral subcortical pathology. The underlying etiologies may include stroke, tumor, trauma, or developmental defects [9, 10, 11, 12, 13]. The concept of ‘bilateral involvement’ encompasses not only structural damage but also functional abnormalities [14]. For example, in some patients with opercular syndrome, head MRI reveals only unilateral structural abnormalities, while SPECT imaging demonstrates functional impairment on the contralateral side [15, 16]. A case report has documented FCMS resulting from bilateral corona radiata infarcts [17], and diffusion tensor tractography has shown reduced volume of the bilateral corticonuclear tracts of FCMS patients [12]. In general, FCMS caused by bilateral opercular damage generally carries a poor outcome, with most patients exhibiting persistent neurological deficits. In contrast, patients with FCMS resulting from unilateral opercular involvement tend to have a relatively favorable prognosis [18, 19]. The fundamental pathophysiology of FCMS involves decompensation of the bilateral corticonuclear tracts, leading to pseudobulbar palsy. Because the corticonuclear tract provides bilateral innervation to the upper facial muscles but crossed innervation to the lower facial muscles, functional impairment is typically more pronounced in the lower face and tongue, predisposing patients to disorders of mastication, swallowing, and articulation.

Thus, our summary indicates that classifying FCMS based on the anatomical distribution of involvement yields five specific subtypes, which can be categorized as either bilateral or unilateral. Bilateral anterior opercular syndrome (Involvement of both the anterior and the frontal operculum) [3, 9]; Opercular‐subopercular syndrome (Opercular cortical involvement on one side with subopercular involvement contralaterally) [12]; Subopercular syndrome (Involvement confined to subcortical corticonuclear projections) [17]; Unilateral anterior syndrome involving the frontal operculum and bilateral leukoaraiosis [10, 11]; Posterior syndrome (Isolated unilateral involvement) [19].

The inability to close the eyelids voluntarily, known as apraxia of eyelid closure, has been associated with damage to the right frontal and parietal lobes [20, 21, 22]. A case series study highlighted the dominant role of the right hemisphere in governing eyelid movements, finding impairments in 23% of the patients with right brain damage compared with only 8% of those with left brain damage. This discrepancy suggests a significant contribution of the right hemisphere to the control of eyelid function [23]. Nevertheless, this is not a definitive criterion, as a similar dissociation has been observed in cases of bilateral disruption of the connections between the cortical motor area and cranial nerve nuclei. Such disruption can occur in conditions like pseudobulbar palsy (specifically opercular syndrome), as well as in Creutzfeldt‐Jakob disease, multiple sclerosis, and amyotrophic lateral sclerosis (ALS) [24].

These impulses then reach the facial nerve, which innervates the bilateral orbicularis oculi muscles, leading to the formation of the ipsilateral R2 and the contralateral R2′ responses. The centers for these reflexes are situated in the pontine tegmentum. Both the R2 and R2′ reflexes are polysynaptic, and because their reflex arcs involve polysynaptic connections within the reticular formation, they are particularly susceptible to influence from the thalamus and cortex [2]. The finding of an absent R2′ response on blink reflex testing provides objective electrophysiological evidence of a brainstem lesion affecting this central polysynaptic pathway. This finding directly corroborates our central thesis by demonstrating functional disruption of the corticobulbar tracts at the pontine level. Researchers have identified a region within the primary motor cortex (area M1) that innervates the upper facial muscles. This area is regulated by inter‐cortical inhibitory and disinhibitory loops and is responsible for generating nerve impulses that reach both the upper and lower portions of the contralateral facial muscles. Infarctions affecting this area can lead to abnormalities in the blink reflex (BR). These findings suggest that cortical damage impacts not only the pyramidal tract but also the facial sensory representation within the cerebral cortex. Consequently, the blink reflex may be more significantly affected by cortical involvement than by basal ganglia pathology [4].

Disorders of voluntary eyelid closure, despite preserved reflex performance, have been occasionally reported after brain lesions and remain subject to varied interpretations. The abnormality may manifest as either a failure to initiate and maintain lid closure or a failure to initiate or maintain lid opening. The former inability was first described by Lewandowsky [25] and has since been repeatedly reported in patients with bilateral [26] and unilateral brain lesions [27, 28, 29], particularly those confined to the right hemisphere. Other patients could close their eyelids but were unable to keep them shut for more than a few seconds, despite repeated prompting. This phenomenon is termed motor impersistence [28, 30].

Acupuncture has been recognized as a safe and viable approach for treating conditions affecting the central and peripheral nervous systems. Numerous studies have confirmed its efficacy in treating blink disorders [31, 32]. Certain studies have highlighted that acupuncture applied to local points around the orbit can stimulate the muscles responsible for eye movement, enhancing muscle activity and alleviating symptoms of paralysis [33, 34, 35]. Additionally, acupuncture at local orbital points has been shown to modulate peripheral microvascular circulation, enhancing blood flow. This leads to improved capillary flow rates and energy metabolism, thereby promoting the recovery of muscle and nerve function around the orbit. In the present case, acupuncture therapy was associated with improvement in the patient's apraxia of eyelid closure, a finding not previously reported in the literature. However, the underlying mechanism requires further investigation, as spontaneous recovery during the natural disease course or the effects of concomitant medication cannot be ruled out.

## 6 Limitations

This study has several limitations. As this is a single case report, we did not perform advanced investigations such as SPECT/PE, diffusion tensor tractography, or evoked potentials (EPs) to further explore the underlying mechanisms. Regarding therapeutic intervention, repetitive transcranial magnetic stimulation (rTMS) shows potential for addressing autonomic‐voluntary dissociation in patients; however, additional clinical trials are required to validate its efficacy. Early recognition of these clinical features is crucial for guiding acute intervention, nutritional support, rehabilitation, and secondary prevention [36, 37]. Objective assessments confirmed significant functional impairment in this patient, supporting a favorable prognosis.

## Author Contributions

**Lu Xia:** conceptualization, writing – original draft, writing – review and editing. **Liyan Cai:** methodology. **Xin Chen:** data curation. **Wei Huang:** methodology. **Cen Chen:** methodology. **Shinian Yang:** methodology. **Qing Chen:** methodology. **Yuanyuan Zhu:** resources.

## Funding

This work was supported by the Research Project of Sichuan Provincial Administration of Traditional Chinese Medicine under Grant No. 25MSZX200 and the Health Commission of Chengdu under Grant No. 2022322.

## Ethics Statement

The study was approved by the Research Ethics Committee of Chengdu First People's Hospital (No. 2024‐YNYJ‐039).

## Consent

The participant provided written informed consent before participating in this study.

## Conflicts of Interest

The authors declare no conflicts of interest.

## Supporting information


**Video S1:** Dynamic laryngoscopy of the Foix‐Chavany‐Marie Syndrome patient. The video demonstrated that the patient has dysphagia during the oral and pharyngeal phases, loose left vocal cords, and closed in an arc shape. Consuming low, medium, and high‐thick foods, residue can be seen in the pyriform sinus and glottic fossa, which can be cleared by repeated swallowing.

**Video S2:** Eyelid Closure Apraxia of the Foix‐Chavany‐Marie Syndrome patient. The video demonstrated the eyelid motor impersistence (MI) and the effectiveness of acupuncture treatment.

## Data Availability

The data that support the findings of this study are available on request from the corresponding author. The data are not publicly available due to privacy or ethical restrictions.
